# Dendritic Actin Cytoskeleton: Structure, Functions, and Regulations

**DOI:** 10.3389/fncel.2017.00147

**Published:** 2017-05-18

**Authors:** Anja Konietzny, Julia Bär, Marina Mikhaylova

**Affiliations:** DFG Emmy Noether Group ‘Neuronal Protein Transport,’ Center for Molecular Neurobiology (ZMNH), University Medical Center Hamburg-EppendorfHamburg, Germany

**Keywords:** dendrites, actin, cytoskeleton, protein trafficking, Arp2/3-complex, formin, cofilin

## Abstract

Actin is a versatile and ubiquitous cytoskeletal protein that plays a major role in both the establishment and the maintenance of neuronal polarity. For a long time, the most prominent roles that were attributed to actin in neurons were the movement of growth cones, polarized cargo sorting at the axon initial segment, and the dynamic plasticity of dendritic spines, since those compartments contain large accumulations of actin filaments (F-actin) that can be readily visualized using electron- and fluorescence microscopy. With the development of super-resolution microscopy in the past few years, previously unknown structures of the actin cytoskeleton have been uncovered: a periodic lattice consisting of actin and spectrin seems to pervade not only the whole axon, but also dendrites and even the necks of dendritic spines. Apart from that striking feature, patches of F-actin and deep actin filament bundles have been described along the lengths of neurites. So far, research has been focused on the specific roles of actin in the axon, while it is becoming more and more apparent that in the dendrite, actin is not only confined to dendritic spines, but serves many additional and important functions. In this review, we focus on recent developments regarding the role of actin in dendrite morphology, the regulation of actin dynamics by internal and external factors, and the role of F-actin in dendritic protein trafficking.

## Introduction

The unique ability of neurons to compute and allocate information relies on their polarized morphology, which comprises several functionally distinct compartments. Dendrites are long, highly branched extensions from the cell body that can reach hundreds of microns, forming a widespread and complex arbor. They integrate information from typically thousands of synaptic inputs, which is then further transmitted via the cell body to the neuron’s single axon (Magee, 2000; Gulledge et al., 2005). Dendrites can be morphologically and functionally sub-compartmentalized, particularly in pyramidal neurons (Shah et al., 2010; Yuan et al., 2015). One of the critical aspects in establishment and maintenance of the dendritic structure is the well-controlled turnover of cytoskeletal elements (Tsaneva-Atanasova et al., 2009). F-actin and microtubules (MTs) are the main mediators of neuronal polarity. Their organization is spatially and temporally controlled by numerous actin binding proteins (ABPs) and microtubule associated proteins, which extensively interact and feed back to each other (Georges et al., 2008; Coles and Bradke, 2015). The process of neuronal polarization is largely driven by an intrinsic program (Horton et al., 2006), however, this program is subject to modification by diverse environmental stimuli, including synaptic activity, that can rapidly feed back to the cytoskeleton. In light of novel discoveries related to the role and organization of neuronal F-actin, in this review we will focus on the mechanisms and molecular players that fine-tune the actin cytoskeleton, thereby controlling dendrite morphology and function.

## Organization of F-Actin in Dendrites

**FIGURE 1:**
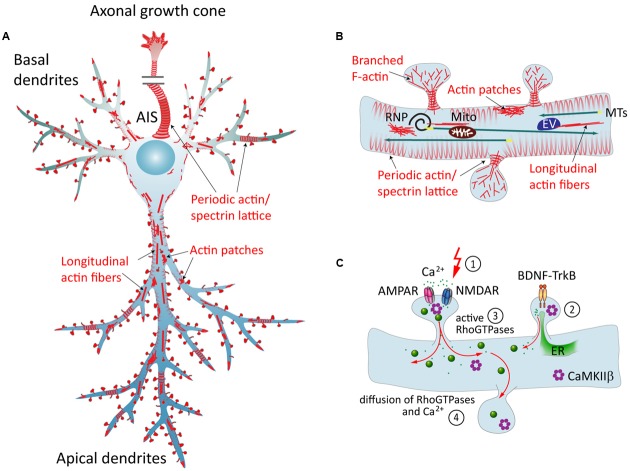
**The neuronal actin cytoskeleton and its regulation by external factors.** **(A)** Overview of different actin structures present in pyramidal neurons: local F-actin enrichments called actin patches, longitudinal actin fibers, and a cortical periodic actin/spectrin lattice termed “actin rings” can be found throughout axon and in dendrites. **(B)** Dendritic spines contain branched F-actin in the head, and straight bundles as well as a periodic actin lattice in the neck. Directed transport of cargo from the soma to the dendrite is carried out via MTs, and can then be subjected to activity-dependent positioning at the base of activated spines in an F-actin and myosin-dependent manner. EV = endosomal vesicle, RNP = ribonucleoprotein, Mito = mitochondrium. **(C)** Dynamics of the dendritic actin cytoskeleton are influenced by external cues. Those include the transduction of external signals to the actin cytoskeleton via cell-surface receptors that couple to Rho-GEFs or ABPs, and Ca^2+^ signaling. The latter involves Ca^2+^ influx through glutamate receptors following synaptic stimulation (1), and Ca^2+^ release from internal stores, triggered for instance by BDNF-TrkB-signaling (2). Both pathways include the activation of Rho-GTPases (3), which act as “molecular switches” that govern a multitude of cellular functions. Diffusible factors, like Ca^2+^, Rho-GTPases, CaMKIIβ and other downstream effectors, can spread the signal from their activation site to the dendrite and to other spines (4). ER = endoplasmatic reticulum.

Actin filaments can be arranged in linear or in branched conformations, and together with stable MT arrays and neurofilaments they form the cytoskeleton in dendrites (Yuan et al., 2012; Sainath and Gallo, 2014). Perhaps the most striking F-actin-based structures in dendrites are so-called spines, small membranous protrusions that harbor synapses. F-actin arrangement within spines is very dynamic and is subject to constant activity-dependent remodeling (Okamoto et al., 2004). Apart from that, additional F-actin based structures within the shafts of dendrites have been discovered more recently: actin patches, longitudinal fibers, and rings (**Figures 1A,B**). **Actin patches** are areas of a few microns enriched in branched F-actin (Willig et al., 2014), and were suggested to serve as outgrowth points for filopodia (Korobova and Svitkina, 2010). **Longitudinal actin fibers** are long bundles of F-actin that traverse along the lengths of dendrites (D’Este et al., 2015; Bär et al., 2016). Their properties and functions are so far unexplored. **Actin rings**, originally described in axons (Xu et al., 2013), are periodic cortical actin structures that are also present in dendrites and in necks of dendritic spines (D’Este et al., 2015; Bär et al., 2016; He et al., 2016). According to the current model, this periodic lattice consists of several short and stable actin filaments, capped by α-adducin, and crosslinked by α/β-spectrin tetramers that define the spacing between the rings (Xu et al., 2013; Qu et al., 2016). These structures are thought to support neurite shape, help in organization of proteins along the plasma membrane (Xu et al., 2013), stabilize the underlying MT cytoskeleton (Qu et al., 2016) and could influence spine neck elasticity during transport of organelles (Bär et al., 2016).

## Regulation of Dendritic Actin Cytoskeleton

### Controlling F-Actin Turnover: Actin Nucleation Factors, Severing and Capping Proteins

Like in any other cell, many functions of actin in neurons relate to its ability to polymerize and depolymerize in response to cellular signaling. Although not specifically studied in dendrites, numerous ABPs are known that cooperate in controlling the structure and stability of F-actin networks and their ability to shape cellular membranes. A summary can be found in **Table 1**, whereas in the text below we will focus on the mechanisms that may be particularly relevant in the regulation of dendritic F-actin.

**Table 1:** Actin binding proteins in neurons and their cross-talk with MTs.

Protein group	Function in neurons	Binding partners	Reference
**Actin monomer (G-actin) binding**			
β-Thymosin	G-actin “buffer,” blocks all known assembly reactions but with a high on/off rate		Yarmola and Bubb, 2004
Profilin	Actin nucleotide exchange factor; maintains G/F-actin ratio together with capping proteins	ROCK, Formin, VASP, WAVE, WASP, Drebrin	Witke et al., 1998; Da Silva et al., 2003
CAP	*Cyclase associated protein*; actin nucleotide exchange factor; sequesters G-actin and severs F-actin; role in growth cone and dendrite development	Profilin, Abp1, Abl, Ras, Cofilin	Ono, 2013; Kumar et al., 2016
**Nucleators**			
Arp2/3-complex	F-actin branching in lamellipodia growth cones, and spine heads	WASP, WAVE, Cortactin	Korobova and Svitkina, 2008; Hotulainen et al., 2009
Formins	Filament nucleation in filopodia, growth cones and along axon; synergize with other actin nucleators	Rho, Rac, Cdc42, Spire, APC	Matusek et al., 2008; Ganguly et al., 2015
Cobl	Role in dendrite branching and growth cones	CaM, Syndapin-1	Ahuja et al., 2007
Spire	Dendrite arborization in *Drosophila* sensory neurons; cooperates with formin-1/2; Myosin V recruitment	Formin-1/2, Myosin Vb	Ferreira et al., 2014; Pylypenko et al., 2016
**Elongation-promoting factors**			
Ena/VASP	Accelerate elongation and prevent capping; role in filopodia formation and neurite elaboration	WAVE, Profilin	Dent et al., 2007; Kwiatkowski et al., 2007; Chen et al., 2014
**Barbed end capping**			
CapZ	Maintains G/F actin ratio together with profilin; role in neurite elaboration		Davis et al., 2009
Adducin	Promotes F-actin bundling and spectrin binding; component of actin rings	Spectrin	Leite et al., 2016
**Pointed end capping**			
Tropomodulins	Stabilize F-actin and decelerate actin dynamics; associated with growth cones	Tropomyosins	Cox et al., 2003; Schevzov et al., 2012
**Crosslinkers/Bundling**			
Fimbrin	Axiogenesis	Spectrin	Oprea et al., 2008
Spectrin	Couples F-actin cytoskeleton to plasma membrane; component of actin rings	Adducin, Fimbrin, α-Actinin	Bennett and Baines, 2001; Xu et al., 2013
α-Actinin	Calcium sensitive; role in dendrite elaboration and branching		Hodges et al., 2014
**Severing**			
ADF/cofilin	Bind and sever F-actin, enhance depolymerization; role in spines and LTP; bind G-actin and enhance nucleation	CaMKII, LIMK, Calcineurin, Slingshot, CAP	Meyer and Feldman, 2002; Andrianantoandro and Pollard, 2006
Gelsolin	Severs F-actin, directly activated by Ca^2+^; role in growth cone and spines	Ca^2+^	Furukawa et al., 1997; Star et al., 2002
**Stabilizing**			
Cortactin	Stabilization of F-actin; activation of Arp2/3; in filopodia and growth cones	Arp2/3, WASP	Kowalski et al., 2005; Korobova and Svitkina, 2008
Abp1	Associates with newly formed, dynamic F-actin; concentrated at subcortical post-synaptic scaffold	Arp2/3, WASP, Cobl	Kessels et al., 2000; Pinyol et al., 2007
Drebrin	Stabilizes actin, competitively inhibits binding of tropomyosins, myosins, fascin and other ABPs; recruits MT into growth cones and dendritic spines	EB3	Geraldo et al., 2008; Merriam et al., 2013
Tropomyosin	Bind along actin filaments; role in dendrite elaboration; effect depends on interaction with other ABPs		Schevzov et al., 2005; Tojkander et al., 2011; Curthoys et al., 2014
**Actin-MT crosslinkers**			
MAP1/2	Ability to crosslink microtubules with F-actin; formation and stabilization of neurites		Roger et al., 2004; Szebenyi et al., 2005
MACF1	*MT-actin crosslinking factor 1,* also known as *ACF7, Shortstop,* and *kakapo;* Role in dendrite branching		Sun et al., 2001; Applewhite et al., 2010
EB3	MT plus-end tracking protein (+TIP); can simultaneously link to actin via drebrin; role during neuritogenesis	Drebrin	Geraldo et al., 2008
APC	*Adenomatous polyposis coli protein*; possesses actin nucleation activity and might crosslink to MT via the +TIP IQGAP	IQGAP, mDia	Watanabe et al., 2004; Okada et al., 2010
Formins	Simultaneous actin- and MT-binding activity; might also crosslink actin and MT via APC:IQGAP	APC	Bartolini et al., 2008; Breitsprecher et al., 2012
CTTNBP2	*Cortactin-binding protein 2,* neuron-specific, possible interactor between cortactin and microtubules	Cortactin	Shih et al., 2014
CLIP170	*EB3-associated +TIP*; might link MT to actin via IQGAP	IQGAP	Swiech et al., 2011
P140Cap	*EB3-associated +TIP*; might link MT to actin via cortactin	Cortactin	Jaworski et al., 2009
Abl	*Ableson-family of non-receptor tyrosine kinases*; bind both actin and MT; activate WAVE complex	WAVE, Ena/VASP	Moresco, 2005; Colicelli, 2010
**Motor proteins**			
Myosin II; with light chains RLC and ELC	Network contraction; F-actin shearing; remodeling of actin network in growth cones and in spines undergoing LTP	MLCK, ROCK, MLCP	Medeiros et al., 2006; Rex et al., 2010; Koskinen et al., 2014

As the rate-limiting step in actin polymerization, nucleation is a crucial point in regulating F-actin dynamics. Several actin nucleators, including the Arp2/3-complex, WASP-homology-2 (WH2) domain proteins and formin-homology (FH) proteins, facilitate this process. The **Arp2/3**-**complex** is the only known regulator for actin branching. It requires an existing actin filament, from which it nucleates a new filament branch (Smith et al., 2013). The Arp2/3-complex is activated by membrane-associated interactors, such as neuronal Wiskott–Aldrich Syndrome protein (N-WASP) or WASP-family verprolin-homologous protein (WAVE) (Korobova and Svitkina, 2010). Arp2/3-complex-dependent polymerization of branched actin networks generates widespread pushing forces against the plasma membrane, accounting for its prominent role in the maturation and enlargement of dendritic spines (Bosch et al., 2014; Spence et al., 2016). Another mechanism of activation involves the F-actin binding protein **cortactin**, which can bind and activate the Arp2/3-complex both directly and indirectly via N-WASP (Kowalski et al., 2005; Korobova and Svitkina, 2008). The Arp2/3-complex and cortactin are enriched in both axonal and dendritic growth cones of young hippocampal neurons (Strasser et al., 2004) and in dendritic spines of mature neurons (Hering and Sheng, 2003). While overexpression of Arp2/3-complex subunits or N-WASP affect both dendrite and axon development, a deficiency of those proteins induces excessive growth and branching exclusively of the axon (Strasser et al., 2004; Pinyol et al., 2007). Dendritic phenotypes seen at the later stages of development are mostly related to attenuated filopodia and spine formation (Spence et al., 2016). The precise molecular mechanisms behind such differential effects have yet to be elucidated (Sainath and Gallo, 2014). Still, it hints at a functional redundancy with other actin nucleators specific to dendrite development. Here, elaboration critically depends on the WH2-domain nucleator **Cobl** (Ahuja et al., 2007), which acts as a positive regulator of neurite outgrowth and branching in rat primary hippocampal neurons (Hou et al., 2015).

**Formins** are actin nucleators downstream of Rho-GTPases (Matusek et al., 2008; Kühn and Geyer, 2014). They nucleate unbranched actin filaments and are mainly associated with the outgrowth of filopodia (Hotulainen et al., 2009). Additionally, they play a role in coordinating MT functions, since they have a distinct MT bundling activity (Bartolini et al., 2008). Formins are involved in proper axon development (Matusek et al., 2008), and in the formation of a deep actin network within the axon, where actin filaments are nucleated from the surface of stationary endosomes in so-called “F-actin hotspots” (Ganguly et al., 2015). Whether the same mechanism is behind the formation of F-actin patches and longitudinal F-actin bundles that have been observed in dendrites (D’Este et al., 2015; Sidenstein et al., 2016) is unknown. Interestingly, another WH2-domain nucleator, **Spire** (Spir-1/2), directly interacts with the formins Fmn-1/2 (Pechlivanis et al., 2009). It was shown that in several non-neuronal cell types, those two proteins are recruited to recycling endosomes and cooperate in the nucleation of F-actin from the vesicle’s surface (Schuh, 2011; Pylypenko et al., 2016). Whether this mechanism is active in neurons has not been investigated so far. However, since the expression patterns of Spire1 and Fmn-2 markedly overlap in the mouse brain (Schumacher et al., 2004), the existence of such a mechanism in neurons seems plausible.

F-actin turnover is greatly accelerated by filament severing proteins, like the closely related **ADF** and **cofilin-1** (Sarmiere and Bamburg, 2004). They increase the number of uncapped ends that may undergo polymerization and regulate the G/F-actin pool (Andrianantoandro and Pollard, 2006). Binding of ADF/cofilin to F-actin additionally induces a conformational change, which can affect binding of other ABPs (Ngo et al., 2016). The activity of ADF/cofilin is tightly regulated via several mechanisms, including phosphorylation (CaMKII, LIMK) and dephosphorylation (calcineurin, slingshot). For a detailed review on ADF/cofilin, see (Kanellos and Frame, 2016). Of note, cofilin-1 activity is instrumental for the dynamic plasticity of dendritic spines (Noguchi et al., 2016), and it is possible that activated cofilin-1 could spread out from a single activated spine to drive re-organization of F-actin in associated dendritic compartments.

### Extracellular Factors Controlling Actin Dynamics in Dendrites

There is a vast number of studies addressing the role of **cell adhesion molecules** (CAMs) and extracellular guidance cues in neuronal cell migration, axon pathfinding, axon-dendrite contact formation and dendritic spine plasticity (Togashi et al., 2009), whereas their role in dendritogenesis has been somewhat overlooked. Those cell surface receptors associate directly with ABPs, thereby translating environmental cues into local changes in actin dynamics (Leshchyns’ka and Sytnyk, 2016).

Neural CAM1 (**NCAM1**) has been extensively studied for its role in neuronal development (Li et al., 2013; Leshchyns’ka and Sytnyk, 2016). It was shown recently that the isoform NCAM180 is highly enriched at dendritic growth cones in rat primary hippocampal neurons during dendritogenesis (Frese et al., 2017). Knockdown of NCAM1 led to reduced dendrite lengths, most likely due to absence of NCAM1-mediated actin stabilization, since many different ABPs were found to be associated with its intracellular domain (Pollerberg et al., 2013; Frese et al., 2017). These results suggest a novel role of NCAM180 in dendritic arborization. Of note, other NCAM family proteins have also been reported to be involved in dendritic branching and morphology in *C. elegans* (Dong et al., 2013).

**Integrins** are another type of surface receptors in direct contact with the actin cytoskeleton. They interact with components of the extracellular matrix (ECM) and affect actin dynamics through associated **Abl-family tyrosine kinases**, of which **Arg** is particularly abundant in the nervous system and localizes to dendritic spines (Lin et al., 2013). Arg controls both dendritic spine and dendrite arbor stability through distinct pathways: it promotes binding of cortactin to F-actin to stabilize spines (MacGrath and Koleske, 2012), and attenuates Rho activity to stabilize dendrite arbors (Moresco, 2005; Lin et al., 2013).

An important feature of mature neurons is dendrite compartmentalization, for example the distinction between apical and basal dendrites, or between proximal and distal regions of apical dendrites. Those compartments are characterized by the expression of specific sets of ion channels (Ginger et al., 2013). Little is known about the mechanisms behind this distinction, however, several studies demonstrated that the large secreted matrix glycoprotein **Reelin** influences positioning of the Golgi apparatus toward the future apical dendrite (Leemhuis and Bock, 2011; Meseke et al., 2013), and that it is required for establishing and maintaining the molecular identity of the distal dendritic compartment of pyramidal neurons (Kupferman et al., 2014). Reelin signals through lipoprotein-receptors, activating both the GSK3β- and PI3K-Rho-GTPase-pathways, which influence the MT and actin cytoskeleton, respectively (González-Billault et al., 2005; Leemhuis and Bock, 2011). Additionally, Reelin-signaling was found to inactivate ADF/cofilin via LIMK, thereby stabilizing F-actin (Chai et al., 2009, 2016).

### Role of Synaptic Activity in Shaping the Dendritic Actin Cytoskeleton

Apart from direct contact with the ECM and neighboring cells, another important factor for dendrite survival and stabilization is synaptic input (Niell et al., 2004). Increased calcium influx via glutamate receptors and L-type Ca^2+^-channels at excitatory synapses stabilizes dendritic branches (Lohmann et al., 2002). In addition, neurotrophic signaling via brain-derived neurotrophic factor (BDNF) modulates calcium signaling. Activation of the TrkB receptor by BDNF triggers multiple downstream pathways, which promote synaptic potentiation but also dendrite growth and stabilization (Horch and Katz, 2002; Wang et al., 2015). The downstream signaling is mediated by the activation of Rac1-GTPase and MAP-kinases, which influence both the actin and microtubule cytoskeleton, and of PLC-γ and PI3-kinase, which trigger the release of calcium from the endoplasmic reticulum (ER) (*reviewed in* Huang and Reichardt, 2003).

Although most of the Ca^2+^-dependent effects have been described in spines, Ca^2+^ diffuses from activated spines and thus can activate dendritic targets (**Figure 1C**). Cytoplasmic Ca^2+^-signaling is largely transduced via the ubiquitous Ca^2+^-sensor calmodulin (CaM), which rapidly activates CaM-kinases and calcineurin (Ca^2+^/CaM-dependent phosphatase). **CaMKII**, at the center of many signaling cascades, regulates formation, growth, and branching of dendrites locally via **Rho-GTPases,** which modulate cytoskeleton turnover, and globally via activation of transcription factors (*reviewed in* Redmond and Ghosh, 2005). Apart from this, CaMKIIβ possesses an F-actin binding ability, enabling the dodecameric holoenzyme to cross-link and stabilize actin networks (Lin and Redmond, 2008; Na et al., 2016). Activated CaMKII is then released from F-actin, which constitutes one of the many ways to link Ca^2+^-signaling to the regulation of the actin cytoskeleton. Several other ABPs are known to be directly influenced by CaM/Ca^2+^, including spectrins and actinin, ADF/cofilin and gelsolin (Oertner and Matus, 2005) and Cobl (Hou et al., 2015). However, there may still be additional, so far unidentified calcium sensors that directly couple Ca^2+^-signaling to actin dynamics.

Numerous ABPs are further indirectly activated downstream of CaM/Ca^2+^ and CaMKII via Rho-GTPases (Boekhoorn and Hoogenraad, 2013). Three well-studied Rho-GTPases that drive cytoskeleton-mediated dendrite morphogenesis are **RhoA, Rac1** and **Cdc42** (Negishi and Katoh, 2002). It was shown that during potentiation of synaptic spines, Rho-GTPases get activated and can then diffuse along the membrane into the dendrite and neighboring spines (Murakoshi et al., 2011). While this kind of “spillover” has been suggested to play a role in clustering of activated synaptic inputs (*discussed in* van Bommel and Mikhaylova, 2016), continued signaling within the dendritic shaft might as well be involved in activity-dependent stabilization of the whole dendrite (**Figure 1C**).

Rho-GTPases activate a myriad of both intertwining and antagonistic pathways that signal to the actin and microtubule cytoskeleton, their effectors including kinases, formins, MAPs, WASP-family proteins and other ABPs. For a detailed review on Rho-GTPases and their role in organizing the actin cytoskeleton, see (Sit and Manser, 2011).

## F-Actin in Transporting and Localization of Cargo within Dendrites

Maintenance of the polarized dendrite morphology does not only depend on the cytoskeletal scaffold, but also on the constant supply of membrane components and dendrite-specific cargo (Hanus and Ehlers, 2016). Long-range intra-dendritic cargo transport is typically carried out via MTs and associated motors. However, actin and actin-dependent motors (myosins) have been shown to mediate the transport and/or anchoring of certain cargos, which include mRNA, translational machinery and mitochondria (Ligon and Steward, 2000; *reviewed in* Martin and Ephrussi, 2010).

For transport, **mRNA** is packaged into ribonucleoprotein particles (RNPs) containing specific targeting factors, and is delivered from the soma to the dendrite via MTs. Some RNPs are targeted to spines in an activity-dependent manner, which requires the presence of F-actin (Huang et al., 2007; Yoon et al., 2016). Likewise, myosin Va (MyoVa) was shown to facilitate the accumulation of RNPs in spines (Yoshimura et al., 2006). As a general model, activity-dependent targeting of cargo to activated synapses has been proposed to involve myosins located at the spine neck, which take up cargo that has been unloaded from passing MT-motors in a Ca^2+^-dependent manner (Hanus et al., 2014). Similarly, it has been shown that dendritic mitochondria show activity-induced movement toward dendritic spines in dissociated neurons. This process likely involves Arp2/3-complex-mediated actin polymerization via mitochondria-associated WAVE1 (Sung et al., 2008). It has been speculated that this mechanism might ensure the local energy supply at sites of activity. However, in dendrites within intact tissues, mitochondria are mainly immobile and localize stably to synapses and branch points (Faits et al., 2016), so the *in vivo* role of this observation is uncertain. In this context, the possibility that actin rings could serve as cargo-docking sites has been brought up, which would allow precise control of mitochondria localization (Gallo, 2013). Supporting this, the speed of axonal mitochondria transport decreased in an α-adducin knockout background, which affects the integrity of actin rings (Leite et al., 2016), and in axons of *Drosophila* neurons, knockdown of MyoV and MyoVI impacts mitochondria transport (Pathak et al., 2010).

Within the actin-rich environment of dendritic spines, myosin motors are known to play an important role in the transport of vesicular cargo (Osterweil et al., 2005; Wang et al., 2008). In cerebellar Purkinje neurons, MyoVa acts as a processive organelle transporter that moves the ER into dendritic spines, which is required for long-term synaptic depression (Wagner et al., 2011). Whether this motor is also involved in the more dynamic spine-localization of ER in other types of neurons, just like the role of myosins in transport and anchoring of dendritic organelles in general, still remains to be explored.

## Concluding Remarks

Our current knowledge of the organization, polarity, and dynamics of actin along dendritic shafts is very incomplete, although the recent development of super-resolution microscopy has provided us with additional tools to study the architecture of the actin cytoskeleton in greater detail. So far, it has led to the discovery of a periodic actin lattice along the lengths of neurites, as well as actin patches and deep actin filaments, whose function and properties are still unexplored. Particularly because of the high degree of conservation among species, it would be interesting to learn more about the mechanisms and nucleation factors involved in formation and regulation of these structures.

Synaptic input and cell contacts play a critical role in the stabilization of dendrites. All of those inputs converge to finally modulate cytoskeleton dynamics, with the main effectors being MTs and F-actin. Thanks to extensive research efforts, a myriad of intertwining pathways and molecular cascades that signal to the cytoskeleton have been described. However, how a given input might lead to an observed output in such a complicated multi-factor system is often hard to reconstruct in detail. Therefore, our understanding of how those different pathways are coordinated and integrated within the cell would greatly benefit from a concerted *in silico* modeling approach. A special interest lies on the question how and to what extent the wealth of described signaling factors that modulate F-actin dynamics within dendritic spines can extend their signaling into dendritic shafts, as a diffusional activity has for example been described for Rho-GTPases and cofilin.

A lot of research regarding mechanisms shaping neuronal actin networks, including their modulation by intrinsic and extrinsic signaling, has been focused on the axon, and here it is important to test whether the identified mechanisms and pathways of F-actin remodeling are applicable to dendrites as well. For example, it will be important to investigate whether dendritic F-actin patches, which at first glance appear to share a similar structure with axonal F-actin “hotspots,” actually originate from stationary endosomes as well, or whether they constitute their own unique features. For now, we conclude that in analogy to the axon, the dendritic actin cytoskeleton might play a two-fold role: stable, cortical actin rings provide mechanical support, while dynamic, underlying filaments sustain physiological processes related to dendritic and synaptic plasticity.

## Author Contributions

AK, JB, and MM wrote the manuscript and all authors commented on the final version.

## Conflict of Interest Statement

The authors declare that the research was conducted in the absence of any commercial or financial relationships that could be construed as a potential conflict of interest.
